# Self‐managed abortions in Ghana: A health policy framework analysis

**DOI:** 10.1002/puh2.101

**Published:** 2023-06-23

**Authors:** Emmanuel Komla Senanu Morhe, Fred Yao Gbagbo, Renee Aku Sitsofe Morhe

**Affiliations:** ^1^ Department of Obstetrics and Gynaecology School of Medicine University of Health and Allied Sciences Ho Ghana; ^2^ Department of Health Administration and Education Faculty of Science Education University of Education Winneba Ghana; ^3^ Department of Private Law, Faculty of Law Kwame Nkrumah University of Science and Technology Kumasi Ghana

**Keywords:** abortion law, Africa, Ghana, maternal health, policy analysis, self‐managed abortion

## Abstract

**Background:**

Self‐managed abortions (SMAs) remain a public health challenge; and are worst in deprived settings. In this policy review, we sought to analyze the legal and policy frameworks within which SMA occurs and look at how these may help improve abortion outcomes in Ghana.

**Methods:**

We searched and reviewed documents on “self‐induced” or “self‐managed” abortion in Ghana from 2015 to 2022. Databases searched included Ghana Digital Attorney, PubMed Central, Google Scholar, and Repositories of Public Universities in Ghana. The key documents reviewed included the abortion law (Act 29) of Ghana, the fourth (2021) edition of the Ghana Health Services’ Comprehensive Abortion Care Standards and Protocols, and the 2017 Maternal Health Survey report. Key documents reviewed included amended Act 29, Comprehensive Abortion Care policy, and standards. We then performed policy analysis using Walt and Gilson's policy triangle framework regarding the context, practice, processes, and key players.

**Results:**

After a careful review of the literature, the following key themes emerged in the framework analysis: the policy environment for SMA, the practice of SMAs, key players of SMAs, consequences of induced abortions, the abortion law, and criminal connotations of SMA. We found that SMAs remain criminalized in Ghana but the local practice persists with the use of registered and unregistered abortifacients. We also observed frequent criminal connotations of SMAs in the literature but no evidence of related prosecutions. There was limited empirical evidence on the safety and efficacy of SMAs in Ghana.

**Conclusion:**

From our findings, we contend that there is an unduly high criminal connotation of SMA in Ghana. We, therefore, recommend a multilevel stakeholder engagement to decriminalize SMAs to ensure improved access to safe abortions in Ghana.

## BACKGROUND

Every day, many women, including adolescents within varied jurisdictions of the world are saddled with taking the right decision in safely resolving unwanted pregnancies that often result from violations of their reproductive rights [1]. Although abortion choice is influenced by a woman's socio‐legal setting and resources, access to quality care is inadequate in many countries due to the negative undercurrents of induced abortion [2], leading to over 25 million unsafe abortions with about 31,000 deaths of women occurring annually [3, 4]. Women in countries that have resisted liberalization of laws on abortion have limited access to care with high levels of self‐managed abortion (SMA) [5]. Even in countries where abortion is technically legal, a myriad of factors make safe abortions rarely accessible. These include lack of public support [6], absence of trained specialists [7], administrative/logistical challenges [8], low economic empowerment of women [9], inadequate information about legal rights to abortion services [10], and abortion‐related stigma [11]. Abortion seekers use SMA to circumvent these challenges [12, 13]. SMA in the current context involves self‐sourcing abortion pills and managing the process outside a healthcare facility [14]. With the appropriate use of recommended medications, SMA is safe and effective [15]. However, successful SMA intervention requires a good flow of accurate information in the community regarding updated law, and policy, relevant processes, and key players.

Ghana's law on abortion is enshrined in the Criminal Offences Act, (Act 29 of 1960, as amended by the 1985 Provisional National Defence Council Law [PNDCL] 102) [16]. Despite the liberalization of the law and development of a policy to provide abortion care to the full extent permitted by law, high rates of “criminal” abortion remain frequently reported in the country [17, 18]. From the 2017 Maternal Health Survey report, about 71% of the most recent abortions were said to be “criminal abortions.” The majority of these were self‐induced abortions [18]. It has been observed that enhancing the use of improved SMA practices reduces abortion complications. The reform is most successful in settings where accurate information is available, medical care for the delivery of support services is readily accessible, and other abortion methods are legal and readily accessible [19]. We sought to analyze the current law, policy, practices, and consequences of SMA and the potential enhancement to improve abortion care in Ghana.

## METHODS

### Design

This was a policy analysis of the current status of SMA in Ghana. We adopted a step‐by‐step framework approach in searching, selecting, and critical review of relevant and reliable documents found in gray and peer‐reviewed literature to unearth key issues of interest to stakeholders in optimizing the practice. We identified gaps in the legal and policy frameworks, which provided a functionally sound and fair basis for discussions and recommendations.

### Data extraction and analysis

Both hard and electronic copies of relevant documents were obtained from the Library of the School of Public Health, University of Ghana, Legon. We also searched the Ghana Digital Attorney for abortion‐related law reports. Other databases searched included PubMed Central, Google Scholar, and Repositories of Public Universities in Ghana. For the electronic search, we narrowed the search to “Ghana” and “SMA” or “self‐induced abortion” “abortion pill self‐medication,” and “self‐prescription of medical termination of pregnancy” as the key search items. We identified fifteen (15) related papers published works in the period 2015–2022 and reported safety, efficacy, and consequences of the practice in Ghana [20].

**FIGURE 1:**
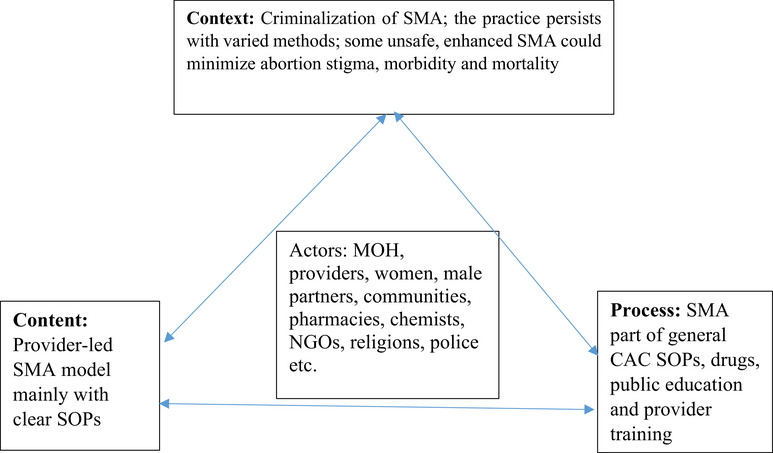
Self‐managed abortion policy analysis in Ghana: Walt and Gilson's policy triangle framework [21].

For the analysis, we employed Walt and Gilson's policy triangle framework [21]. This analytical framework has been prepared specifically for health policy analysis. It is simple and focuses on four key areas of health policy: the policy context, content, process, and key actors that are critical to the success of any health reform (Figure 1). We performed content analyses of the abortion law of Ghana [16] and the Comprehensive Abortion Care (CAC) Policy, Standards and Protocols in Ghana [17]. We looked specifically at aspects of the revised CAC Policy, standards and Protocols published in January 2021 in relation to the previous editions to examine the progress being made in policy formulation and implementation with respect to CAC in Ghana.

## RESULTS

The key documents reviewed included the abortion law (Act 29) of Ghana, the fourth (2021) edition of the Ghana Health Services’ Comprehensive Abortion Care Standards and Protocols, and the 2017 Maternal Health Survey report. The key themes that emerged from framework analyses of these documents include the law and criminal connotation of SMA, the policy environment, prevailing practices, and key actors of SMAs.

### Criminal connotations of self‐managed abortions

Self‐induced/SMA is prohibited by the Ghanaian Criminal Offences Act (Act 29), section 58, sub‐section 1 [16]. The law states that (a) “a woman who, with intent to cause abortion or miscarriage, administers to herself or consents to be administered to her a poison, drug or any other noxious thing or uses an instrument or any other means, or (b) a person who: (i) administers to a woman a poison, drug or any other noxious thing or uses an instrument or any other means with the intent to cause abortion or miscarriage of that woman, whether or not that woman is pregnant or has given her consent, (ii) induces a woman to cause or consent to causing abortion or miscarriage, (iii) aids and abets a woman to cause abortion or miscarriage, (iv) attempts to cause abortion of miscarriage, or (v) supplies or procures a poison, drug, an instrument or any other thing knowing that it is intended to be used or employed to cause abortion or miscarriage; commits a criminal offense and is liable on conviction to a term of imprisonment not exceeding five years.” [16] Abortion is permissible under certain situations, as stated in section 2 of the statute. These situations include when the pregnancy results from rape, defilement, or incest; when the continuation of the pregnancy would involve risk to the life of the pregnant woman or injury to her physical or mental health; or where there is a substantial risk that if the pregnancy was carried to term the child would suffer from or later develop a serious physical abnormality or disease [16]. The section is also emphatic on allowing gynecologists or other registered medical practitioners to provide abortion care in registered health facilities. The abortion Law of Ghana is, however, silent on SMAs.

### Policy environment for self‐managed abortions

Based on the permissive provisions in sub‐section 2 of the law, the national CAC policy, standards, and protocols were developed with the broad operational interpretation of the law [18]. The main policy that was developed and incorporated into national reproductive health policy was to provide abortion care to the full extent permitted by law. The purpose was to minimize abortion‐related maternal morbidity and mortality in the country [18]. Hence, CAC has been made an integral part of the national reproductive health policy and program. With a task‐sharing strategy, the CAC policy allows various cadres of healthcare professionals to provide both surgical and medication abortions [17].

The policy standards have been revised progressively in line with World Health Organization (WHO) recommendations and the need to improve the safety of induced abortion in Ghana. Thus, CAC services have become increasingly available to women in need. In the current (fourth, 2021 edition) policy standards, three options for the administration of medical abortion in the first trimester are indicated. Based on the client's eligibility, the options can be home‐based administration (self‐care) or health facility, and home‐based administration or facility‐based administration [17]. The protocols are clear; for gestations below 9 weeks, the pregnant woman can take the medication and has expulsion at home after receiving clear guidance from a healthcare provider [17]. The criteria for home‐based SMA and circumstances, under which women being managed at home can seek help at healthcare facilities, are clearly outlined. Only healthcare facilities with established links and provide all components of CAC are recommended to provide support for SMA users. The practice of SMA without healthcare professional guidance is not recommended [17].

### Key actors of self‐managed abortions

Both provider‐led and in‐person SMAs are practiced in Ghana. Notwithstanding the legal prohibitions, self‐induced abortion with medications has become the leading choice for many abortion care seekers in Ghana [18]. Over the years, many women have used over‐the‐counter medications, herbal concoctions, and other locally known chemical abortifacients. Some of these preparations have been widely reported to be associated with severe maternal complications [22, 23].

In line with current evidence, there has been an increasing shift to the use of misoprostol‐based medications that may be cheaper alternatives to hospital‐procured abortion methods [22, 24]. The 2017 Maternal Health Survey Report indicated that about three in four (73%) of the most recent induced abortions were by misoprostol/mifepristone preparations [18]. Currently, misoprostol only or misoprostol–mifepristone combination is the commonest method of self‐induced abortion among educated and other well‐informed women in Ghana [24]. There are a number of key actors in enhancing SMA. These include lawmakers and enforcers; policymakers and implementers, such as health facilities, individual health care providers, women with unwanted pregnancies, and their aides. Other factors that influenced abortion decision‐making in Ghana include circumstances surrounding the onset of pregnancy [25], the influence of the male partner and other role players [26], the woman's socioeconomic status and self‐efficacy [27, 28].

Both trained and untrained providers are often involved in SMA in varied settings in the country. Reported locations of medication abortions include public and private health facilities, homes, pharmacies, or chemists. Many women rely on pharmacies and chemists for supplies and guidance on how to use the abortion pill. There are also reported disparities in urban, rural, or urban‐slam misoprostol demand and supply at local pharmacies and chemists. Despite the wide distribution of community pharmacies and chemists, high demand for the pill, local stocking, and willingness to sell the medication decreased from urban to rural settings [18].

The perception that abortion is illegal, social stigma, negative provider attitudes, and the need to keep the unwanted pregnancy and the abortion process secret promotes home‐based SMA [29, 30]. Cost is another main reason for recourse to SMA or use of the services of untrained personnel not indicated in the national policy standards as providers [22, 27]. Trained abortion services may be available but the cost may be beyond most women, particularly the poor, the youth, and other socially disadvantaged women [29, 30–32]. Second‐trimester abortions, for instance, are very expensive and are mostly available in a few private health facilities in urban centers [33]. Reports indicated that most providers are aware of the fact that if women had no access to safe abortion services, they would resort to unsafe methods of terminating unwanted pregnancies [34].

### Consequences of induced abortions

The Ghana Maternal Health Survey Report indicated medical complications and social stigma as the most widely reported consequences of induced abortions [18]. However, the report did not indicate the proportion of the complications emanating from SMA. The gap was partly attributed to poor documentation, and the unwillingness of clients to disclose their real experiences for fear of societal stigmatization, shame, embarrassment, and criminalization [35, 36, 37]. Indeed, a study has shown that much more is yet to be understood about SMA in Ghana: methods used, safety, effectiveness, client experiences, and reasons for the choice of the approach to resolving unwanted pregnancy [23]. We found no evidence of prosecutions of a person(s) found violating the abortion law of Ghana in the review.

## DISCUSSION

The key documents examined in this paper were Act 29, section 58, the fourth (2021) edition of the Ghana Health Service CAC Policy, Standards and Protocols, and the 2017 Maternal Health Survey Report. The paper ascribes to the operational definition of SMA by the WHO as the act of self‐management of the process of medication abortion. In line with current evidence, we fall in with the view that access to improved SMA practices should be seen as empowering women, and an active extension of the essential healthcare system via task sharing [38].

From the 2017 Ghana Maternal Health Survey Report and other publications, it is apparent that women with unwanted pregnancies use a variety of methods for SMA. The use of unsafe methods is associated with complications especially in deprived settings where health services may not be readily available. This underscored the earlier opposition from the developed world [39] when the WHO SMA documents were being developed [38]. Indeed, the uses of medications have become a leading and convenient option for many abortion seekers in Ghana [23]. This gives credence to the need to promote the use of safer methods to reduce abortion‐related complications in the country [18].

Albeit the criminalization of self‐induced abortion, it is prudent and strategic to include SMA in the fourth (2021) edition of the Ghana Health Service CAC Policy Standards and Protocols [17]. We observed that although self‐induced abortion has been extensively reported in Ghana, there are limited related empirical studies on the outcomes of the process in Ghana. Additionally, we also believed that although WHO has provided comprehensive guidelines on SMA [40], local empirical evidence is required to inform policy.

Like other nations, abortion decision‐making in Ghana is influenced by various factors. These include circumstances surrounding the onset of the index pregnancy [25], the male partner and other role players [26], and ultimately the woman's socioeconomic status and self‐efficacy [27, 28]. Indeed, within reproductive rights in the national constitution, the abortion care seeker in Ghana could claim justification to end unwanted pregnancy as indicated in the CAC standards and protocols which give direction to how the law should be operationalized [16, 17, 18, 23, 25, 40, 41].

The abortion law of Ghana, however, imposes penalty on the pregnant woman who seeks abortion care and individual(s) who influence(s) or help her undergo the procedure. We believe this stance of the law could promote abortion stigma and resort to SMA in communities. Hence, to protect the life and health of vulnerable women, SMA needs to be safe and readily accessible to those in need at local settings. It is a trite knowledge that the safety and effectiveness of SMA depend on accurate information flow in the community particularly recommended methods, sources of supply, and when to seek help during the process. This emphasizes the importance of health education in policy formulation and implementation. In this case, comprehensive sex education particularly, in schools, is paramount to ensuring that young people receive accurate and simple information on reproductive health.

Indeed, most people, including some providers, are not aware of the details of the legal status of induced abortion in Ghana and often exhibit attitudes that negatively affect access to safe abortion care in the country [41, 42]. Similar observations have been made in India and Zambia, where abortion is legal, yet unsafe abortions still prevail due to misinformation about the law [19]. By policy, Ghana has gone far by ensuring that in giving consent for induced abortion in Ghana, the decision is predominantly a prerogative of the pregnant woman. The protocols clearly indicate that spousal involvement is encouraged for marital harmony but not mandatory [24].

The WHO clearly indicated in its updated Abortion Care Guideline [40] that medical abortion can be safely and effectively provided by mid‐level health care providers as well as women themselves through telemedicine up to 9 weeks gestation but did not emphasize major complications that are rarely associated with SMAs outside the clinical environments at such low gestations. There was, however, little evidence found on complications associated with the use of telemedicine and digital means for abortion despite the increasing demand and access to SMA services [43, 44]. As indicated by other researchers [45, 46], advocating for SMAs requires expanding the task sharing of abortion care initiatives to include mid‐level providers who will also facilitate accurate information flow in the community to enhance the use of SMA safely. In Ghana, as the revised abortion standards and protocols make provision for self‐managed abortion [17], acceptability of task‐sharing abortion care with mid‐level providers to enhance access to safe abortion care through SMAs may be acceptable, as evidence exists that outcomes are found to be similar if the medical abortion is offered by doctors, nurse midwives, or administered by women themselves [40].

As observed in some communities in Ghana, the criminalization of self‐induced abortion in Ghana has not reduced the practice but rather promoted social stigma and inadequate access to provider‐based services with women resorting to unsafe methods or patronage of services of untrained personnel [47, 48, 49]. With the availability of improved and safer abortion methods, the review of the abortion law should be considered to create an enabling environment where abortion rights are respected and SMA care can be provided through a telemedicine‐based approach to provide accurate information and enhance confidentiality and privacy. Despite the seemingly favorable legal and policy environment, access to safe abortion care remains a challenge to most service seekers in Ghana partly due to financial costs [22]. With a focus on task‐sharing abortion care, SMA could reduce the costs of induced abortions and improve access to safe abortion care among poor and socially deprived populations [47, 49].

## LIMITATIONS

The study could be limited by our understanding of the law, policy, and the development of analytical frameworks as the interpretations and views of the policymakers, lawmakers, and other researchers in this area of study could vary significantly.

## CONCLUSIONS

There is a misalignment of the law and CAC policy standards regarding SMAs in Ghana. Despite the criminalization of SMA, the practice persists in varied forms without records of any prosecutions. Considering the potential of improved SMA in reducing abortion stigma and complications, we contend that there is an unduly high criminal connotation to SMA in Ghana. We recommend multilevel stakeholder engagement on SMA, decriminalize the practice, and consider it as an effective medicolegal intervention to enhance access to safe abortion in Ghana. Empirical research on the safety, efficacy, acceptability, and cost‐effectiveness of SMAs in Ghana is needed.

## AUTHOR CONTRIBUTIONS

Emmanuel Komla Senanu Morhe conceptualized the study from a medical perspective. Fred Yao Gbagbo developed the outline of the study and drafted the manuscript. Renee Aku Sitsofe Morhe reviewed the medicolegal implications of the concepts within the Ghanaian context. Desk review, analysis, and interpretations of information were done by all authors. Emmanuel Komla Senanu Morhe, Fred Yao Gbagbo, and Renee Aku Sitsofe Morhe, respectively, reviewed the manuscript critically for medical, policy, and legal accuracies. Fred Yao Gbagbo developed the final draft and did the editing and correspondence with the publishers. All authors have read the final manuscript and have consented to its publication.

## CONFLICT OF INTEREST STATEMENT

We declare that we have no conflict(s) of interest in this study.

## FUNDING INFORMATION

The study received no external funding. The authors covered all expense related to this paper.

## ETHICS STATEMENT

Ethics approval was not applicable to this study since the study did not involve human participants.

## Data Availability

The raw data and any material related to the study are available upon reasonable request from the corresponding author.
